# Data on structural transitions in domains of hordeivirus TGB1 protein forming ribonucleoprotein complex

**DOI:** 10.1016/j.dib.2016.05.012

**Published:** 2016-05-14

**Authors:** Valentin V. Makarov, Svetlana S. Makarova, Natalia O. Kalinina

**Affiliations:** a Belozersky Institute of Physico-Chemical Biology, Lomonosov Moscow State University, Leninsky Gory, Moscow 119992, Russia; b Department of Virology, Lomonosov Moscow State University, Leninsky Gory, Moscow 119992, Russia

**Keywords:** Hordeivirus, RNP-complexes, Plant virus transport

## Abstract

This data article is related to the research article entitled “*in vitro* properties of hordeivirus TGB1 protein forming ribonucleoprotein complexes” (Makarov et al., 2015 [1]), demonstrating that upon incubation with viral RNA the poa semilatent hordeivirus (PSLV) TGB1 protein (the movement 63 K protein encoded by the first gene of the triple gene block) *in vitro* forms RNP structures resembling filamentous virus-like particles and its internal domain (ID) performs a major structural role in this process. This article reports the additional results on the structural lability of ID and the structural transitions in the C-terminal NTPase/helicase domain (HELD) induced by interaction with tRNA and phosphorylation.

**Specifications Table**

**Table:** 

Subject area	*Biology*
More specific subject area	*Structural virology*
Type of data	*Figures*
How data was acquired	*CD and absorption spectra, electrophoresis image*
Data format	*Analyzed*
Experimental factors	*Isolation of recombinant proteins, measurement of CD and absorption spectra on Chirascan CD and Hithachi UV-2900 instruments*
Experimental features	*Structural transitions in deletion mutants of hordeivirus TGBp1*
Data source location	*Belozersky Institute of Physico-Chemical Biology and Department of Virology, Lomonosov Moscow State University, Leninsky Gory, Moscow, 119992, Russia*
Data accessibility	*Data are provided with this article*

**Value of the data**

• The data show that structural conversion (increasing of *β*-structure content) in the TGBp1 internal domain (ID) is initially induced by interaction with RNA and leads to protein multimerization/aggregation. These data further demonstrate the importance of β-structure for RNA-protein and protein-protein interactions.
• The data demonstrate that the ID phosphorylation [2] is accompanied by the domain secondary structure transition to a predominantly disordered state and may explain common mechanisms of RNP complex destabilization.
• The increasing in the content of the β-component upon incubation RNA with HELD is mainly due to the structural transitions in the NTPase sub-domain displaying RNA-binding activity [3]. These data may be of interest for studying rearrangement of SF1 helicase structure induced by interaction with RNA.

## 1 Data

An essential step for realization of hordeivirus TGBp1 structural functions such as formation or remodeling/destabilization of transport RNP particles is based on the protein secondary structure conversion [1].

In this article, we display additional data on structural transitions in the internal domain (ID) and the NTPase sub-domain of HELD as a result of their interactions with RNA or phosphorylation.

## 2 Experimental design, materials and methods

### 2.1 Isolation of recombinant proteins

**Fig. 1:**
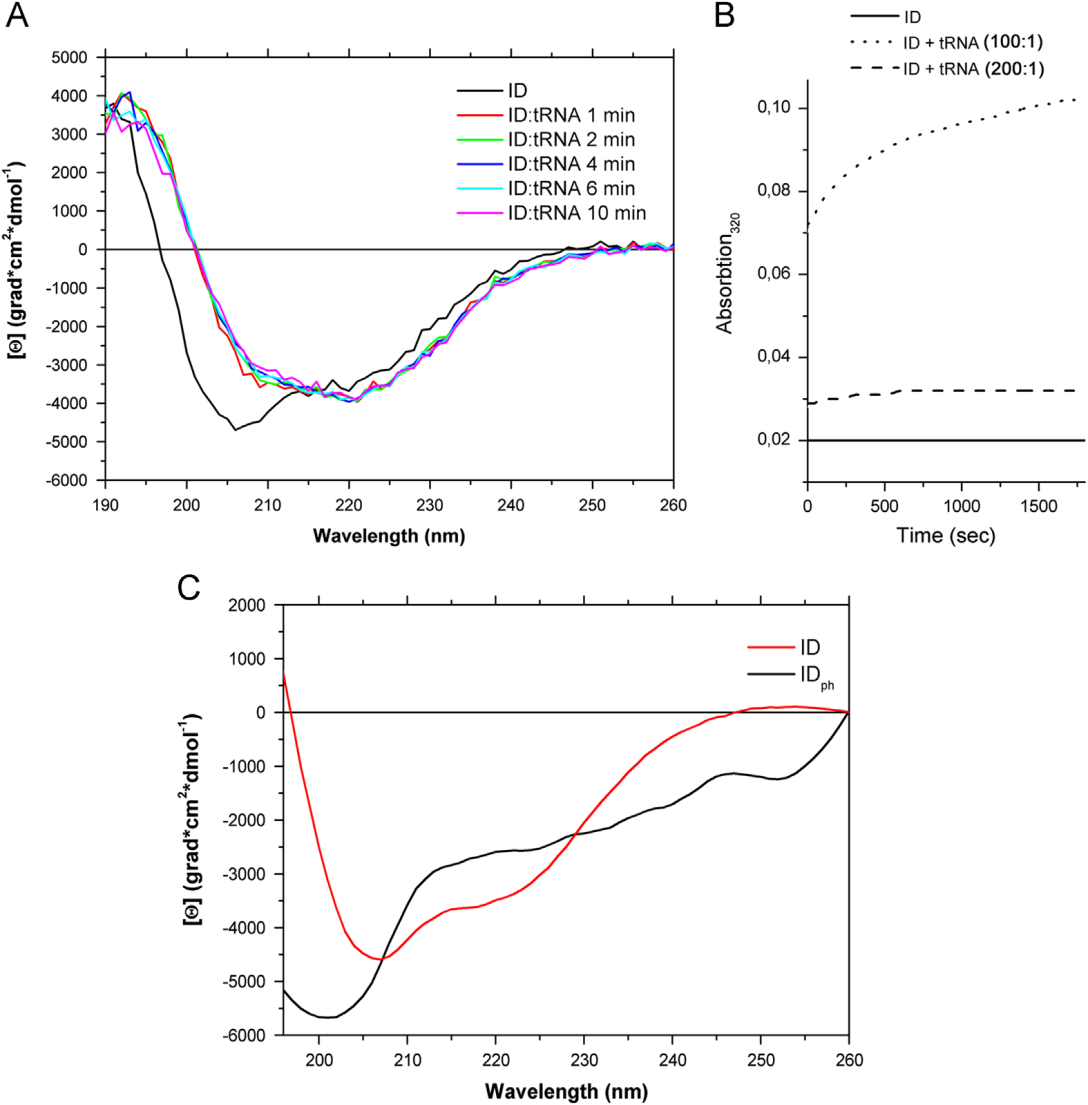
Changes in circular dichroism (CD) spectra of the internal domain (ID) of PSLV TGBp1 in the presence of tRNA or after ID phosphorylation. Far-UV CD spectra of the recombinant proteins recorded at 25 °C. (A) CD spectra of ID at molar protein:RNA ratio 100:1 recorded during different time intervals; (B) kinetic of ID multimerization/aggregation (C) CD spectra of non-phospholylated ID and ID after phosphorylation by protein kinases associated with plant cell walls [2].

**Fig. 2:**
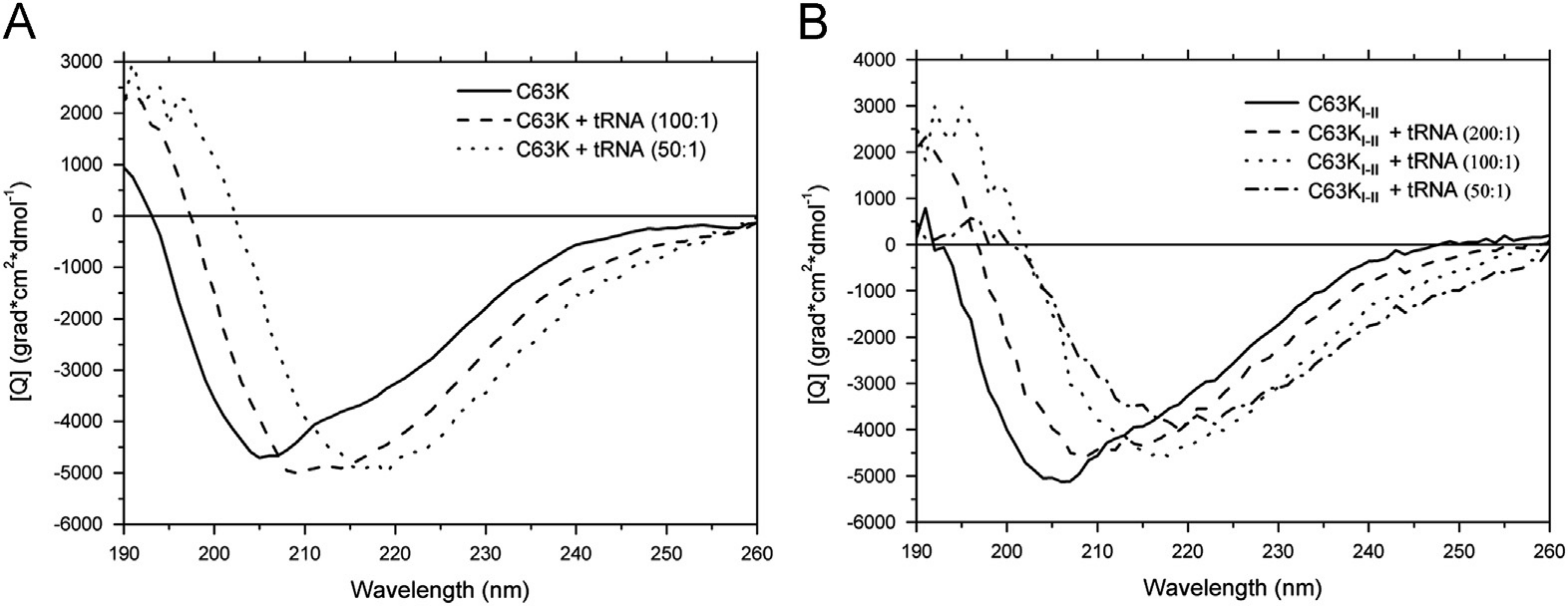
Circular dichroism (CD) spectra of the C-terminal NTPase/helicase domain (HELD) of PSLV TGBp1 (С63K) and the NTPase sub-domain (C63K_I–II_) without and in the presence of tRNA at different molar protein:RNA ratios. Far-UV CD spectra of the recombinant proteins recorded at 25 °C. (A) C63K; (B) C63K_I–II_.

Expression of recombinant protein genes in *Escherichia* *coli* cells, their isolation and purification of (His)_6_ recombinant proteins were performed as described previously [3], [4]. Mutants of the PSLV TBGp1 (63K protein) are indicated according to their previous designations [3], [4] (Fig. 1, Fig. 2).

### 2.2 Measurement of the CD and absorption spectra

Protein samples at the concentration of 100 μg/ml in 1 mM HEPES buffer pH 7.0 were loaded into 1–2-mm cells, and CD spectra were recorded from 185 to 260 nm at 25 °C in a Chirascan CD spectrometer (Applied Photophysics, England). The CD spectra were recorded at rate 0.5–1.0 nm/s with base-line subtraction. The measured spectra were smoothed using the instrument software. The [θ] value calculations were based on mean amino acid residue molecular weight of 110. Absorption spectra were measured in 1 cm cells on Hithachi UV-2900 (Hitachi, Japan).
